# The Impact of Hyperglycemia on Risk of Severe Infections during Early Period of Induction Therapy in Patients with Newly Diagnosed Multiple Myeloma

**DOI:** 10.1155/2014/413149

**Published:** 2014-04-16

**Authors:** Sung-Hoon Jung, Hee-Chang Jang, Seung-Shin Lee, Jae-Sook Ahn, Deok-Hwan Yang, Yeo-Kyeoung Kim, Hyeoung-Joon Kim, Je-Jung Lee

**Affiliations:** ^1^Department of Hematology-Oncology, Chonnam National University Hwasun Hospital, 322 Seoyang-ro, Hwasun, Jeollanam-do 519-763, Republic of Korea; ^2^Department of Infectious Disease, Chonnam National University Hwasun Hospital, 322 Seoyang-ro, Hwasun, Jeollanam-do 519-763, Republic of Korea

## Abstract

The association between hyperglycemia and infections during induction chemotherapy has been reported in a number of hematologic disorders. This retrospective study evaluated the incidence of hyperglycemia during induction therapy in 155 patients with newly diagnosed multiple myeloma (MM) and its effect on serious infections during the first 60 days of induction. A total of 20 (12.9%) patients developed overt hyperglycemia (≥200 mg/dL) during induction therapy. Serious infections occurred in 28 (18.1%) of 155 patients and infection-related mortality within 2 months after treatment was 0.6% (1 patient). In a univariate analysis, overt hyperglycemia, poor performance status (≥2), International Staging System III, lymphopenia (<500/**μ**L), and elevated serum creatinine (≥2 mg/dL) were found to be associated with serious infections. In multivariate analysis, only overt hyperglycemia (HR 7.846, 95% CI 2.512–24.503, *P* < 0.001) and poor performance status (HR 5.801, 95% CI 1.974–17.050, *P* = 0.001) remained significant. In conclusion, this study demonstrated an association between hyperglycemia and serious infections during induction therapy in patients with MM.

## 1. Introduction

Infection is a major cause of morbidity and mortality in patients with multiple myeloma (MM). The increased susceptibility to infection results from the interplay between antineoplastic therapies and age- and disease-related complication [1]. In a retrospective study evaluating the incidence of infection throughout the disease course in patients with MM, nearly half of the patients experienced at least one clinically significant infection in 2 months of initial chemotherapy [2]. Furthermore, one study reported that up to 10% of newly diagnosed MM patients died of infectious cause within 60 days of their diagnosis [3]. These early infections are serious problem in management of MM, but there were few studies to examine the risk factor for early infections in patients with MM.

Corticosteroid is a major treatment agent as a single agent or in combination with other agents in patients with MM. Treatment with steroid could cause hyperglycemia regardless of presence of diabetes mellitus, because it increases peripheral insulin resistance and glucose production and suppresses insulin production [4–6]. Steroid induced hyperglycemia has been reported as risk factor for poor clinical outcomes in patients with hematologic malignancies. A retrospective study reported that patients with acute leukemia having blood glucose levels above 200 mg/dL during chemotherapy including oral dexamethasone have shorter complete remission duration and poor survival outcomes and are more likely to develop infection over the next 10 years than control patients [7]. Other reports also reported that hyperglycemia is associated with increased mortality in patients with acute myeloid leukemia, and increased risk of severe sepsis in hyperglycemic group seems to be partly responsible for the increased mortality [8]. These data suggested that steroid induced hyperglycemia may be an important risk factor for the development of severe infection in patients with MM.

In this study, we evaluated the incidence of hyperglycemia and its association with development of severe infection during early period of initial chemotherapy in patients with MM. We did not include patients with diabetes mellitus to focus on steroid induced hyperglycemia and infection risk in this study.

## 2. Methods

### 2.1. Patients

We retrospectively analyzed the records of 362 patients with newly diagnosed MM between November 2002 and February 2013 at Chonnam National University Hwasun Hospital. We excluded 82 patients who did not have available clinical and laboratory data at diagnosis and follow-up. Twenty-four patients were excluded if they had an active infection during the 7 days prior to initiation of chemotherapy. We also excluded 80 patients who received prophylactic antibiotics during first-line chemotherapy and 21 patients who had diabetes mellitus at diagnosis. Of the patients with MM, 155 were included in this study.

### 2.2. Measurements and Definitions

Fingerstick glucose levels were monitored at least two times a day during hospitalization for initial diagnosis: fasting glucose and 2-hour postprandial glucose. If patients showed high glucose levels during steroid containing induction therapy, fingerstick glucose levels were monitored more often (up to seven times per day). Blood glucose levels were taken at a consistent time points. Glucose levels were measured by ACCU-CHEK Inform II (Roche, Mannheim, Germany). Plasma blood glucose level was also checked in patients treated with further chemotherapy at outpatient clinic. Patients were stratified into three groups by World Health Organization criteria [9] and glucose levels obtained in any time points were used to classify patients into three groups. Mild hyperglycemia was defined as blood glucose 140–200 mg/dL on ≥2 days; overt hyperglycemia was defined as blood glucose ≥200 mg/dL on ≥1 day; all other patients were considered to have euglycemia.

The infection was defined as clinically documented (CDI) when there were clinical signs and symptoms of infection but no pathogen was isolated. If a pathogen was isolated from a blood sample or culture of any site, it was defined as microbiologically documented (MDI). The National Cancer Institute Common Terminology Criteria (NCI-CTC) for Adverse Events (version 4.0) were used to grade infectious complication. Grades 3-4 infections were classified as severe infections. A grade 3 infection was defined as a severe infection, systemic infection requiring intravenous antibiotics, antifungal, or antiviral intervention. A grade 4 infection was defined as life-threatening consequences. When severe infection developed within 60 days of induction chemotherapy, we defined it as early severe infection.

### 2.3. Statistical Analyses

The univariate analysis of factors associated with severe infection was performed using the *χ* ^2^ test. Among the factors, those with *P* < 0.05 were selected and included in the multivariate logistic regression analysis. *P* < 0.05 was considered significant for all analyses. All statistical computations were performed using SPSS software package version 18.0 (SPSS Inc., Chicago, IL, USA)

## 3. Results 

### 3.1. Patient Population

The median age was 61 years (range, 38–81) and 34.2% of patients were ≥65 years. Of all patients, 78 (50.3%) were male. A total of 82 patients (52.9%) had MM of the IgG type and 24.5% had light-chain disease. Performance status of 0, 1, and ≥2 was found in 24, 98, and 33 patients, respectively. With regard to the International Staging System, 49 patients were stage I, 48 were stage II, and 58 were stage III. Overweight (body mass index (BMI): 25–29.9 kg/m^2^) and obese (BMI ≥ 30 kg/m^2^) patients were 24.5% and 4.5%, respectively. All patients received steroid containing regimen as first-line chemotherapy. Twenty-one patients (13.5%) received prednisone, and 134 patients (86.5%) received dexamethasone. A total of 131 patients (84.5%) received a thalidomide-based regimen, such as cyclophosphamide, thalidomide, and dexamethasone (CTD), melphalan, prednisolone, and thalidomide (MPT), and thalidomide and dexamethasone (TD). Ten patients (6.5%) were treated with high-dose dexamethasone only. Five patients (3.2%) received a bortezomib-based regimen. One patient (0.6%) received a combination of lenalidomide and low-dose dexamethasone. Eight patients (5.2%) were treated with other conventional chemotherapies including vincristine, adriamycin, and dexamethasone (VAD), melphalan and prednisolone (MP), and cyclophosphamide with prednisolone (CP) or dexamethasone (CD).

### 3.2. Incidence of Severe Infections

Severe infections occurred in 28 (18.1%) of 155 patients, defined as CDI in 25 (16.1%) and as MDI in 3 (1.9%). Among the severe infections, the most common was pneumonia, which occurred in 20 patients (71.4%). Median time to early severe infection was 20 days (range, 4–57). Three different organisms were found in patients who experienced bacteremia or fungemia:* Streptococcus pneumoniae* (1),* Enterobacter aerogenes* (1), and* Candida parapsilosis* (1). Most of the infections were cured with broad-spectrum antibiotics or antifungal agent therapy, but one patient died due to severe infection within 2 months after treatment. There were no patients who inserted central venous catheter before development of early severe infections.

### 3.3. Glycemic Status and Infection

A total of 20 patients (12.9%) developed overt hyperglycemia during the first 60 days of first-line chemotherapy. Forty-four patients (28.4%) were with mild hyperglycemia and 91 patients (58.7%) were with euglycemia. Severe infection was more common in overt hyperglycemic group rather than mild hyperglycemic and euglycemic group (Table 3). There was no significant association between steroid formulation and overt hyperglycemia (9.4% in prednisone versus 13.4% in dexamethasone, *P* = 1.000).

The univariate analysis revealed that five factors were significantly associated with early severe infection (Table 1): blood sugar (≥200 mg/dL), Eastern Cooperative Oncology Group (ECOG) performance status ≥2, International Staging System III, lymphopenia <500/*μ*L, and serum creatinine ≥2 mg/dL. In multivariate analysis, only overt hyperglycemia (HR 7.846, 95% CI 2.512–24.503, *P* < 0.001) and poor performance status (HR 5.801, 95% CI 1.974–17.050, *P* = 0.001) remained significant (Table 2). Additionally, there was no statistical difference in the development of severe infections according to the formulation of steroid (5.0% in prednisone versus 20.1% in dexamethasone, *P* = 0.126).

## 4. Discussion

Introduction of stem cell transplantation and novel antimyeloma agents, including thalidomide, bortezomib, and lenalidomide, has improved the outcome of patients with MM [10, 11]. These advances have transformed myeloma into a chronic condition. However, infections still remain the significant cause of morbidity and mortality in patients with MM [3]. Infections may occur at the rate of 1.46–4.68 infections per patient-year over the course of MM [2, 12]. Especially, the incidence of infections is 2-3 times higher during the first two months of initial chemotherapy. These early infections may be fatal and were reported as a leading cause of early death during the induction therapy. In addition, early infections frequently lead to substantial delays and dose reduction in subsequent chemotherapy with increased risk of treatment failure [2]. There were well-known various factors that contribute to the increasing risk of infection including immunoparesis, the placement of vascular catheters, type of therapy applied, extent of prior therapy, and presence of comorbidities and organ dysfunction [1]. The use of steroid also affects the development of infection. Dexamethasone-based regimens decrease in cell-mediated immunity and could increase the risk of infection by encapsulated bacterial organisms, viruses, or fungi [13]. Hyperglycemia induced by steroid could play a role of increased susceptibility of infection [14]. However, for hyperglycemia as well as other risk factors for infection, it is not known how much they are contributing to the early occurrence of the infection.

In this retrospective study, we demonstrated that MM patients in overt hyperglycemic group have significantly higher rates of severe infection rather than mild hyperglycemic and euglycemic group during 2 months of initial chemotherapy. This result is consistent with a previous study of patients with adult or childhood acute lymphoblastic leukemia [7, 15]. Although the use of systemic steroid may increase the risk of infection through effects on innate and acquired immunity, some studies support that acute hyperglycemia itself affects all major components of innate immunity and impairs the ability of the host to combat infection [16]. Acute hyperglycemia reduced neutrophil activity such as chemotaxis, formation of reactive oxygen species, and phagocytosis of bacteria, despite the accelerated migration of leukocytes into peripheral tissue [17–20]. Whether there is a threshold glucose level for impaired immunity is unclear. In vitro study suggested that a threshold glucose concentration of 250 mg/dL results in impaired phagocytic function and bactericidal activity of polymorphonuclear leukocytes from nondiabetic patients [21].

While most of risk factors for infection such as immunoparesis, comorbidities, and performance status are irreversible, hyperglycemia induced by steroid could be reversible. A study showed that tight glucose control reduced infection risk in hyperglycemic neurosurgical patients [22]. Strict blood glucose control with intensive insulin therapy also has been shown to reduce morbidity and mortality among critically ill patients in a surgical intensive care unit [23]. However, it is still unclear whether strict glycemic control improves outcome in patients with MM. Some trial did not show significant difference in mortality in intensive glycemic control group [24, 25].

There were some differences in our study compared to previous studies. Patients with overt hyperglycemia during chemotherapy were smaller than previously reported (19.9% versus 58%). This may be due to the lower percentage of obese patients (3.9%) in our study. In addition, glucose levels were not checked in a standardized fashion and there was no standard for glucose control because of retrospective nature of this study. Therefore, the number of patients with overt hyperglycemia may be underestimated. In addition, we did not evaluate incidence of infections by dose of steroid, because the number of patients was relatively small and they received various induction regimens.

In conclusion, overt hyperglycemia during early period of initial chemotherapy was associated with increased risk of severe infection in patients with MM. Further prospective studies are required to evaluate the clinical outcomes in patients with MM.

## Figures and Tables

**Table 1:** Univariate analysis of risk factor for early severe infections during induction therapy (*n* = 155).

Variable	Infection rates 18.1% (28/155)	*P* value	OR (95% CI)
Gender			
Male	17.9% (14/78)	1.000	1.016 (0.448–2.303)
Female	18.2% (14/77)		
Age			
<65 years	21.6% (22/102)	0.129	0.464 (0.176–1.227)
≥65 years	11.3% (6/53)		
ECOG performance status			
<2	12.3% (15/122)	0.001	4.637 (1.918–11.211)
≥2	39.4% (13/33)		
Immunophenotype			
Others	19.7% (23/117)	0.470	0.619 (0.218–1.761)
Light chain	13.2% (5/38)		
Body mass index			
<25 kg/m^2^	20.9% (23/110)	0.174	0.473 (0.168–1.334)
≥25 kg/m^2^	11.1% (5/45)		
ISS			
I-II	10.3% (10/97)	0.002	3.915 (1.658–9.242)
III	31.0% (18/58)		
Serum creatinine, mg/dL			
<2.0	14.0% (17/121)	0.022	2.926 (1.210–7.073)
≥2.0	32.4% (11/34)		
Blood sugar during induction therapy			
<200 mg/dL	12.6% (17/135)	<0.001	8.484 (3.068–23.460)
≥200 mg/dL	55.0% (11/20)		
Early response (after 2 cycles)			
≥PR	15.0% (15/100)	0.196	1.754 (0.765–4.021)
<PR	23.6% (13/55)		
Neutropenia during induction therapy			
≥1,000/*μ*L	17.6% (22/125)	0.793	1.170 (0.428–3.201)
<1,000/*μ*L	20.0% (6/30)		
Lymphopenia during induction therapy			
≥500/*μ*L	12.9% (14/108)	0.022	2.848 (1.229–6.600)
<500/*μ*L	29.8% (14/47)		
Treatment with novel agents			
Yes	18.9% (26/137)	0.531	1.874 (0.405–8.660)
No	11.1% (2/18)		

ECOG: Eastern Cooperative Oncology Group; ISS: International Staging System; PR: partial response.

**Table 2:** Multivariate analysis of risk factors for early severe infections during induction therapy (*n* = 155).

	*P* value	HR (95% CI)
Blood sugar ≥200 mg/dL	<0.001	7.846 (2.512–24.503)
ECOG performance status ≥2	0.001	5.801 (1.974–17.050)
ISS III	0.089	2.654 (0.862–8.171)
Lymphopenia <500/*μ*L	0.981	1.014 (0.334–3.077)
Serum creatinine ≥2 mg/dL	0.444	1.560 (0.500–4.870)

ECOG: Eastern Cooperative Oncology Group; ISS: International Staging System.

**Table 3:** Rates of severe infections between blood sugar groups during induction therapy (*n* = 155).

Blood sugar groups	Infection rates	*P* value	OR (95% CI)
<140 mg/dL	10.9% (10/91)		1 (reference)
140–200 mg/dL	15.9% (7/44)	0.422	1.532 (0.541–4.341)
≥200 mg/dL	55.0% (11/20)	<0.001	9.900 (3.299–29.709)

## References

[1] Nucci M, Anaissie E (2009). Infections in patients with multiple myeloma in the era of high-dose therapy and novel agents. *Clinical Infectious Diseases*.

[2] Perri RT, Hebbel RP, Oken MM (1981). Influence of treatment and response status on infection risk in multiple myeloma. *The American Journal of Medicine*.

[3] Augustson BM, Begum G, Dunn JA (2005). Early mortality after diagnosis of multiple myeloma: analysis of patients entered onto the United Kingdom Medical Research Council trials between 1980 and 2002—Medical Research Council Adult Leukaemia Working Party. *Journal of Clinical Oncology*.

[4] Ranta F, Avram D, Berchtold S (2006). Dexamethasone induces cell death in Insulin-secreting cells, an effect reversed by exendin-4. *Diabetes*.

[5] Rizza RA, Mandarino LJ, Gerich JE (1982). Cortisol-induced Insulin resistance in man: impaired suppression of Glucose production and stimulation of Glucose utilization due to a postreceptor defect of Insulin action. *The Journal of Clinical Endocrinology and Metabolism*.

[6] Coderre L, Vallega GA, Pilch PF, Chipkin SR (1996). In vivo effects of dexamethasone and sucrose on Glucose transport (GLUT- 4) protein tissue distribution. *American Journal of Physiology—Endocrinology and Metabolism*.

[7] Weiser MA, Cabanillas ME, Konopleva M (2004). Relation between the duration of remission and hyperglycemia during induction chemotherapy for acute lymphocytic leukemia with a hyperfractionated cyclophosphamide, vincristine, doxorubicin, and dexamethasone/methotrexate-cytarabine regimen. *Cancer*.

[8] Ali NA, O’Brien JM, Blum W (2007). Hyperglycemia in patients with acute myeloid leukemia is associated with increased hospital mortality. *Cancer*.

[9] Gabir MM, Hanson RL, Dabelea D (2000). The 1997 American Diabetes Association and 1999 World Health Organization criteria for hyperglycemia in the diagnosis and prediction of diabetes. *Diabetes Care*.

[10] Kumar SK, Rajkumar SV, Dispenzieri A (2008). Improved survival in multiple myeloma and the impact of novel therapies. *Blood*.

[11] Brenner H, Gondos A, Pulte D (2008). Recent major improvement in long-term survival of younger patients with multiple myeloma. *Blood*.

[12] Vesole DH, Oken MM, Heckler C (2012). Oral antibiotic prophylaxis of early infection in multiple myeloma: a URCC/ECOG randomized phase III study. *Leukemia*.

[13] Klein NC, Go CH, Cunha BA (2001). Infections associated with steroid use. *Infectious Disease Clinics of North America*.

[14] Khardori R, Adamski A, Khardori N (2007). Infection, immunity, and hormones/endocrine interactions. *Infectious Disease Clinics of North America*.

[15] Dare JM, Moppett JP, Shield JP (2013). The impact of hyperglycemia on risk of infection and early death during induction therapy for acute lymphoblastic leukemia (ALL). *Pediatric Blood & Cancer*.

[16] Turina M, Fry DE, Polk HC (2005). Acute hyperglycemia and the innate immune system: clinical, cellular, and molecular aspects. *Critical Care Medicine*.

[17] Wierusz-Wysocka B, Wysocki H, Wykretowicz A, Klimas R (1988). The influence of increasing Glucose concentrations on selected functions of polymorphonuclear neutrophils. *Acta Diabetologica Latina*.

[18] Alexiewicz JM, Kumar D, Smogorzewski M, Klin M, Massry SG (1995). Polymorphonuclear leukocytes in non-Insulin-dependent diabetes mellitus: abnormalities in metabolism and function. *Annals of Internal Medicine*.

[19] Dhindsa S, Tripathy D, Mohanty P (2004). Differential effects of Glucose and Alcohol on reactive Oxygen species generation and intranuclear nuclear factor-*κ*B in mononuclear cells. *Metabolism: Clinical and Experimental*.

[20] Perner A, Nielsen SE, Rask-Madsen J (2003). High Glucose impairs superoxide production from isolated blood neutrophils. *Intensive Care Medicine*.

[21] Bagdade JD, Root RK, Bulger RJ (1974). Impaired leukocyte function in patients with poorly controlled diabetes. *Diabetes*.

[22] Ooi YC, Dagi TF, Maltenfort M (2012). Tight glycemic control reduces infection and improves neurological outcome in critically ill neurosurgical and neurological patients. *Neurosurgery*.

[23] van den Berghe G, Wouters P, Weekers F (2001). Intensive Insulin therapy in critically ill patients. *The New England Journal of Medicine*.

[24] van den Berghe G, Wilmer A, Hermans G (2006). Intensive Insulin therapy in the medical ICU. *The New England Journal of Medicine*.

[25] Brunkhorst FM, Engel C, Bloos F (2008). Intensive Insulin therapy and pentastarch resuscitation in severe sepsis. *The New England Journal of Medicine*.

